# Conserved roles of *ems/Emx* and *otd/Otx* genes in olfactory and visual system development in *Drosophila* and mouse

**DOI:** 10.1098/rsob.120177

**Published:** 2013-05

**Authors:** Sonia Sen, Heinrich Reichert, K. VijayRaghavan

**Affiliations:** 1 National Centre for Biological Sciences, Tata Institute of Fundamental Research, UAS-GKVK Campus, Bellary Road, Bangalore 560065, India; 2 Biozentrum, University of Basel, Klingelbergstrasse 50/70, 4056 Basel, Switzerland

**Keywords:** evolutionary conservation, sensory systems, empty spiracles, orthodenticle, *Emx1/2*, *Otx1/2*

## Abstract

The regional specialization of brain function has been well documented in the mouse and fruitfly. The expression of regulatory factors in specific regions of the brain during development suggests that they function to establish or maintain this specialization. Here, we focus on two such factors—the *Drosophila* cephalic gap genes *empty spiracles* (*ems*) and *orthodenticle* (*otd*), and their vertebrate homologues *Emx1/2* and *Otx1/2*—and review novel insight into their multiple crucial roles in the formation of complex sensory systems. While the early requirement of these genes in specification of the neuroectoderm has been discussed previously, here we consider more recent studies that elucidate the later functions of these genes in sensory system formation in vertebrates and invertebrates. These new studies show that the *ems* and *Emx* genes in both flies and mice are essential for the development of the peripheral and central neurons of their respective olfactory systems. Moreover, they demonstrate that the *otd* and *Otx* genes in both flies and mice are essential for the development of the peripheral and central neurons of their respective visual systems. Based on these recent experimental findings, we discuss the possibility that the olfactory and visual systems of flies and mice share a common evolutionary origin, in that the conserved visual and olfactory circuit elements derive from conserved domains of *otd/Otx* and *ems/Emx* action in the urbilaterian ancestor.

## 2. Introduction

Nervous system development in *Drosophila* re-employs developmental control genes that initially pattern the early embryo. One class of such early patterning genes is the cephalic gap genes. These are among the earliest zygotic genes to be transcribed during embryogenesis in *Drosophila*. They are first expressed in the anterior region of the blastoderm-stage embryo, under the control of maternal genes, and are essential for the segmentation and identity of the embryonic head segments [1–5]. Mutational inactivation of these early patterning genes results in specific gap-like deficits in the cephalic anlagen that are due to the inability to specify particular head segments [1–3,6–8]. Consequently, structures that normally derive from these head segments are deleted upon the loss of the corresponding cephalic gap genes [8].

In addition to this early embryonic requirement of cephalic gap genes in the specification of head segments, it has emerged in recent years that these ‘early patterning genes’ are also required later in *Drosophila* nervous system development. Prominent examples of this are identified in recent studies that investigate the multiple roles that cephalic gap genes play in the formation of the peripheral and central elements of complex sensory systems.

Nervous system development in *Drosophila* occurs during two phases; the larval nervous system is generated during embryogenesis and the adult nervous system is generated primarily during postembryonic development [9,10]. Correspondingly, complex sensory systems, such as those involved in olfaction and vision, are essentially created twice during development, once during embryogenesis for the larva and once again during postembryonic development for the adult fly. Remarkably, cephalic gap genes act during both phases of nervous system development.

In this review, we consider two cephalic gap genes (*orthodenticle*, *otd* and *empty spiracles*, *ems*) and their requirement in the development of sensory systems. We first focus on *ems*, and review recent studies that reveal a requirement of this cephalic gap gene in multiple aspects of the development of the olfactory systems in both larval and adult *Drosophila*. Subsequently, we focus on *otd*, and review recent studies that show that this cephalic gap gene has important multiple roles in the development of the visual systems of the *Drosophila* larva and adult. In both cases, we also review data that demonstrate comparable requirements for the vertebrate homologues of these genes, *Emx1/2* and *Otx1/2*, in the development of the olfactory and visual systems of murine rodents, which have olfactory and visual circuits that are strikingly similar to those seen in flies. Finally, in the light of these similarities, we discuss the possible developmental and evolutionary significance of this shared requirement for cephalic gap gene action in the formation of complex sensory systems in invertebrates and vertebrates.

## 3. *Ems/Emx* genes are required in the olfactory systems of flies and mice

### 3.1. Ems in the development of the *Drosophila* larval olfactory sensory system

**Figure 1.**
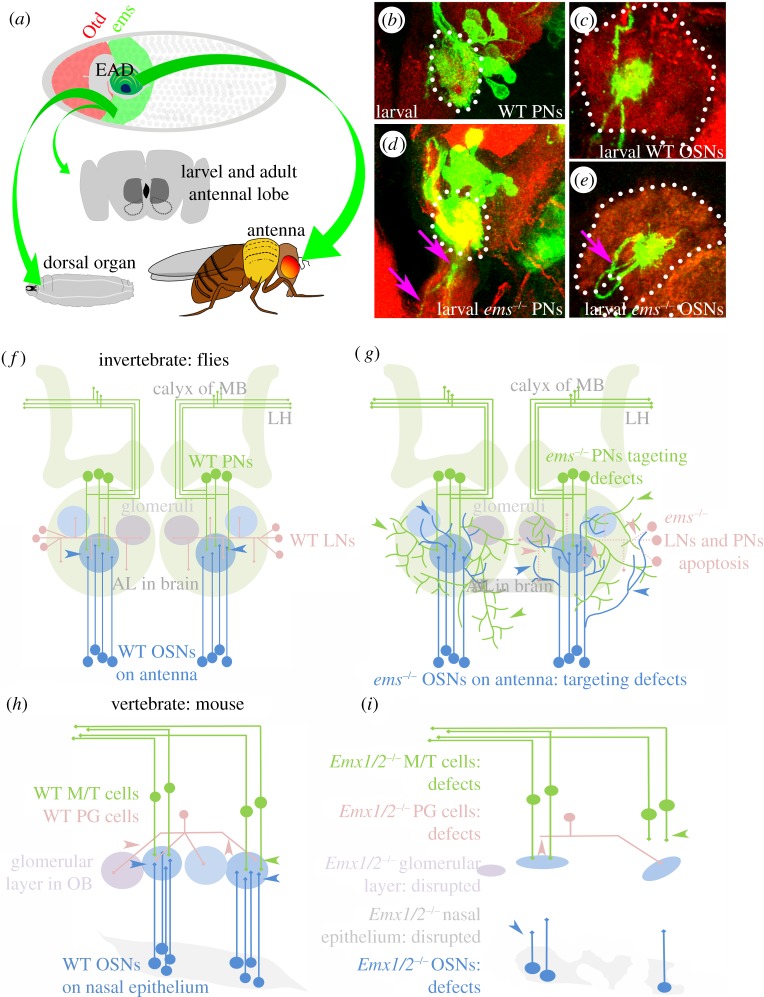
*ems/Emx* genes control the development of the olfactory system in flies and mice. (*a*) The origins of the olfactory neurons in *Drosophila* larvae and adults can be traced back to the Ems-expressing antennal head segment (green stripe in the anterior of the embryo). The larval dorsal organ originates in this segment (green arrow on the left). The neuroblasts that give rise to the deutocerebral larval antennal lobe (grey dotted lines in the brain) and the deutocerebral adult antennal lobe (dark grey shaded area in the brain) delaminate from this Ems-expressing antennal head segment (middle green arrow). The eye–antennal disc (EAD), the antennal part of which gives rise to the adult antenna, also incorporates into it cells from the Ems-expressing antennal head segment (green arrow on the right). (*b*) The WT larval PNs innervating the larval antennal lobe and restricting their dendrites to the confines of the larval antennal lobe (white dotted line). (*c*) The WT OR-45a expressing OSN with terminals confined to a single glomerulus in the antennal lobe (white dotted line). (*d*) The PNs are null for *ems* function and have innervations leaving the antennal lobe (magenta arrows; compare with PNs in (*b*)). (*e*) The OR-45a expressing OSN are null for *ems* function and they have targeting defects (magenta arrow; compare with OSN in (*c*)). (*f*,*h*) Compare the similarity in the olfactory circuits of flies and mice—OSNs (blue neurons) target glomeruli (coloured circles) in the antennal lobe (AL)/olfactory bulb (OB), glomerular specific PNs/mitral–tufted cells (green neurons) take olfactory information to higher brain centres and LNs/periglomerular cells (pink neurons) perform local information processing between glomeruli. (*g*,*i*) Summary of the mutant phenotypes observed in the OSNs, PNs and LNs in flies and mice, respectively, when these neurons/structures are null for *ems/Emx* function. *ems* null fly OSNs fail to respect glomerular and antennal lobe boundaries (blue arrowheads; compare blue neurons in (*f*,*g*)). *ems* null fly PNs from one neuroblast also fail to respect glomerular and antennal lobe boundaries (green arrowheads; compare green neurons in (*f*,*g*)). *ems* null fly LNs and PNs from another neuroblast undergo apoptosis (compare pink neurons in (*f*,*g*)). *Emx* null mice have disrupted nasal epithelia and fewer OSNs, which are unable to target the olfactory bulb (compare blue arrowheads and neurons in (*h*,*i*); also compare nasal epithelium in (*h*,*i*)). The mitral–tufted cells (green neurons) and the periglomerular cells (pink neurons) also fail to target the glomerular layer (compare green and pink arrowheads in (*h*,*i*)), which is also disrupted (compare glomerular layers in (*h*,*i*)).

During embryonic development of the *Drosophila* head neuroectoderm, the cephalic gap gene *ems* is expressed in a broad, stripe-like anterior domain, where it acts in the specification of the so-called antennal segment of the head [1,3]. The *ems*-expressing region of the cephalic neuroectoderm gives rise to the precursors of both the peripheral and the central elements of the larval olfactory system (figure 1*a*).

The major larval olfactory sense organ is the dorsal organ, which contains sensilla that are innervated by the dendrites of 21 olfactory sensory neurons (OSNs). These peripheral sensory neurons target the larval antennal lobe in the deutocerebrum of the central brain, where they synapse onto the olfactory interneurons, the projection neurons and local interneurons, in regions of dense synapses called ‘glomeruli’ [11]. Ems is expressed in the region of the cephalic neuroectoderm that gives rise to the sensory organ precursors of the dorsal organ OSNs, and to the neural stem cell-like precursors, called neuroblasts, which generate the larval olfactory interneurons [8,12].

Mutant analysis shows that *ems* is required for the early embryonic specification of the antennal segment neuroectoderm, from which both the peripheral and central elements of the larval olfactory system derive [8,12]. However, embryos that lack *ems* function are embryonic lethal, and this initially prevented investigation of possible later roles of *ems* in the development of olfactory neurons. More recently, using techniques such as mosaic analysis with a repressible cell marker [13] that allows the generation of *ems* null mutant cells in an otherwise heterozygous animal, and hence circumvents embryonic lethality, several studies have found that *ems* continues to act in the later development of the larval olfactory system. Thus, removal of *ems* from the larval-specific OSNs and interneurons (after generation by their respective precursors) results in the mistargeting of some of these neuronal cells to the larval antennal lobe; sensory neuron terminals are unable to restrict their dendrites to individual glomeruli (cf. figure 1*c*,*e*), and interneuron terminals are seen outside the antennal lobe (cf. figure 1*b*,*d*). These experiments indicate that in addition to the previously documented role that *ems* plays in the early specification of specific subsets of larval OSNs and larval olfactory neuroblasts, *ems* is additionally required later in embryonic development for correct neurite targeting in both of these developing neural cell types in the larval brain.

### 3.2. Ems in the development of the *Drosophila* adult olfactory sensory system

The peripheral and central elements of the adult olfactory system are generated postembryonically and are largely different from the larval olfactory circuit elements. The primary peripheral olfactory sensory structure of the adult *Drosophila* is the antenna. The third segment of the antenna contains approximately 500 sensillar sense organs that are innervated by the dendrites of 1200 adult OSNs. These adult-specific OSNs are different from the larval OSNs since they derive from sensory organ precursors in the eye–antennal imaginal disc during early pupal life [14]. The axons of the OSNs project to and terminate in the deutocerebral region of the adult brain where they make synaptic contacts with the dendrites of projection neurons and local interneurons (figure 1*f*). The projection neurons relay sensory information from the antennal lobe to higher brain centres, and the local interneurons, whose terminals are confined to the antennal lobe, are responsible for local processing of olfactory information [14]. Both types of adult-specific olfactory interneurons are generated during a second, postembryonic wave of neurogenesis by the same deutocerebral neuroblasts that produce the larval-specific olfactory interneurons [15–17]. Hence, apart from the few embryonic born interneurons that persist through metamorphosis [18], all the adult-specific projection neurons and local interneurons are generated postembryonically. It is noteworthy that the precursors for both the peripheral and the central olfactory circuit elements of the adult are lineally related to cells of the antennal head segment; the antenna represents the appendage of the antennal segment and the deutocerebrum of the adult brain corresponds to the neuromere of the antennal segment (figure 1*a*) [12,19].

Recent studies that have examined the postembryonic expression and function of *ems* have found that this embryonically acting cephalic gap gene is also involved postembryonically in the development of the adult-specific olfactory system. Thus, Ems is expressed in a subset of the adult-specific sensory organ precursors and in two of the deutocerebral neuroblasts that produce adult-specific olfactory interneurons [20,21]. In the sensory organ precursors, Ems expression is seen as a short pulse during early pupal life [21]. In the neuroblasts, Ems is expressed throughout larval life and  also transiently expressed in the interneurons that derive from these neuroblasts [16,20]. Mutant analyses show that *ems* is required for the development of the peripheral and central olfactory system (figure 1*g*). In the peripheral olfactory system, *ems* is involved in specification of specific sensillar sense organs and also has a later role in axonal targeting of the OSNs, which derive from these sense organs, to their appropriate antennal lobe glomeruli [21]. In the central olfactory system, the clonal inactivation of *ems* from one of the neuroblasts results in the apoptosis of the interneurons that derive from it, whereas a similar inactivation of *ems* from another neuroblast results in interneurons that fail to innervate their respective glomeruli correctly [16,20]. Thus, in the former case, the antennal lobe has far fewer projection neurons (PNs) and local interneurons (LNs), and is therefore reduced in size, whereas in the latter case the PNs fail to restrict their dendrites to the confines of a given glomerulus, and on occasion even have innervations outside the antennal lobe.

Taken together, these findings show that *ems* has multiple roles in the development of peripheral and central olfactory sensory elements of the larval and adult olfactory systems. Might the vertebrate homologues of *ems*, the *Emx1/2* genes, also have multiple roles in the development of peripheral and central neuronal elements of the mammalian olfactory system?

### 3.3. The mouse olfactory circuit, which shares similarities with flies, requires the *ems* homologues, *Emx1/2*, for development

A striking feature of the olfactory system in insects is the similarity in basic circuit organization that it shares with the mammalian olfactory system [22,23]. Thus, as in *Drosophila*, in the mouse olfactory system, OSNs express odorant receptor genes in a mutually exclusive manner, and the axons of those OSNs that express a given receptor converge onto the same glomerulus. Moreover, in the glomeruli, OSNs make synaptic contacts with primary output interneurons, the projection neurons in the fly and the mitral–tufted cells in the mouse, as well as with local interneurons/periglomerular cells that interconnect glomeruli (figure 1*h*). The layout of the fly and mammalian olfactory circuitry therefore is essentially the same.

In addition, there are remarkable similarities in the expression and function of *ems* and its mammalian homologues *Emx1/2*. Thus, the mammalian *Emx1/2* genes are expressed during early embryonic development in the olfactory placodes and developing nasal epithelium, as well as in the developing forebrain, including the olfactory bulb. (*Emx1/2* genes are also expressed in other areas of the brain that are known to be involved in olfactory processing [24–27].) Mutants for *Emx1/2* have severe defects in the various brain regions, including those involved in olfaction (figure 1*i*). The nasal epithelium, where the cell bodies of the OSNs reside, is disrupted. The axons of the OSNs form a normal olfactory nerve; however, this nerve does not form connections with the olfactory bulb, implying that the OSNs are unable to target correctly. The olfactory bulb of these mutants is extremely reduced in size, and the mitral cell layer of the olfactory bulb is disorganized [28–32]. In addition to these morphological defects, *Emx2* mutants manifest aberrant expression of various odorant receptor genes [33].

In summary, similar to the fly *ems* gene, the mammalian *Emx1/2* genes are expressed in the developing peripheral and central olfactory systems, and are crucial for their proper development. Could other cephalic gap genes play comparable key roles in the development of sensory systems?

## 4. *Otd/Otx* genes are required in the visual systems of flies and mice

### 4.1. Otd in the development of the *Drosophila* larval visual sensory system

**Figure 2.**
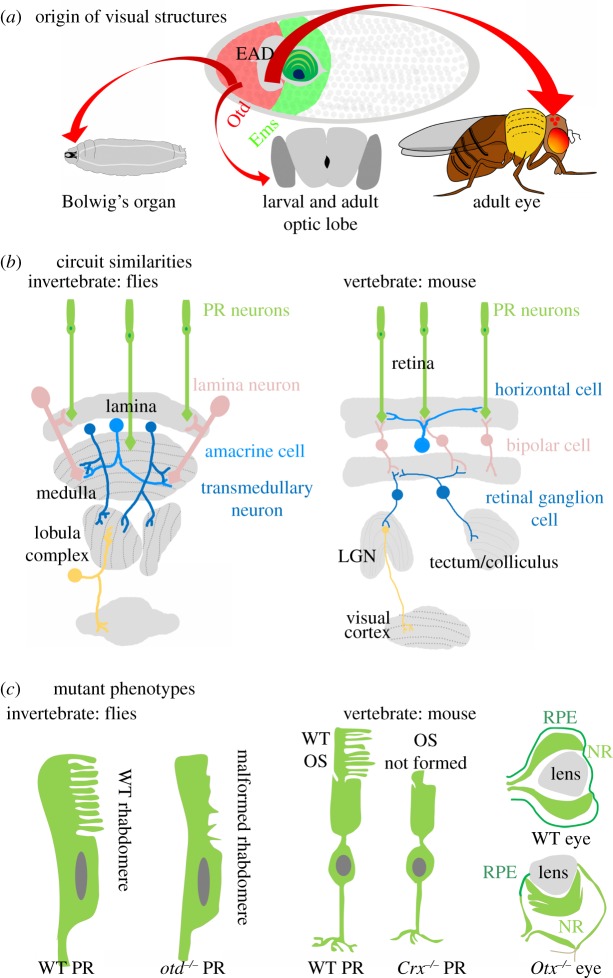
*otd/Otx* genes control the development of the visual system in flies and mice. (*a*) The origins of the visual neurons in *Drosophila* larvae and adults can be traced back to the Otd-expressing ocular head segment (red stripe in the anterior of the embryo). The larval Bolwig's organ originates in this segment (red arrow on left). The optic lobe of the larva and the adult also originate in this segment (middle red arrow). The eye–antennal disc, which gives rise to the adult eye, also incorporates into it cells from the Otd-expressing domain (red arrow on right). (*b*) The similarity in the visual circuits of flies and mice. Photoreceptor cells in both flies and mice (green neurons) project in parallel to a number of interneuronal types (pink and blue neurons). The interneurons are arranged in multiple parallel cell layers (grey structures) that are interconnected orthogonally. (*c*) The mutant phenotypes observed in photoreceptor neurons in *otd* null flies and *Crx* null mice. Note that in both cases, the rhabdomere/outer segment of the PR neurons fails to develop (compare WT and *otd^−/−^* PRs in flies, and WT and *Crx^−/−^* PRs in mice). Also summarized is the phenotype seen in the eyes of mice null for *Otx* function. Note the change in orientation of eye structures and also the expansion of the neural retina at the expense of the retinal pigment epithelium in the *Otx* null mice. EAD, eye–antennal disc; PR, photoreceptor; LGN, lateral geniculate nucleus; RPE, retinal pigment epithelia; NR, neural retina; OS, outer segment.

During embryonic development of the *Drosophila* head neuroectoderm, the cephalic gap gene *otd* is expressed in a broad domain anterior to that of *ems* gene expression where it is thought to act in the specification of the so-called ocular segment of the head [7,8]. This Otd-expressing region gives rise to the cells of the peripheral and central larval visual system (figure 2*a*).

The major larval visual organ is Bolwig's organ, a simple paired structure of 12–14 photoreceptor neurons that extend their axons towards the larval optic neuropile and brain [34,35]. Two distinct sets of central interneurons are postsynaptic to the larval photoreceptor neurons: three optic lobe pioneer neurons that derive from the optic placode, and four pigment dispersing factor (PDF) expressing lateral neurons that are part of a central protocerebral brain lineage [35–37]. During embryogenesis, Otd is expressed in the developing photoreceptor neurons of Bolwig's organ, and this expression is maintained in these sensory cells throughout their larval and adult life [38].

Mutant analysis indicates that *otd* has both early and late roles in the development of Bolwig's organ. In *otd* null mutant embryos, early specification of Bolwig's organ does not occur and hence photoreceptor cells are not formed [8,39]. Hypomorphic *otd* alleles reveal an additional later requirement for the gene in the correct expression of rhodopsin (Rh) subtypes in the differentiating larval photoreceptors. Thus, whereas in the wild-type (WT) eight of the larval photoreceptors express Rh5 and the other four express Rh6, in the *otd* mutants all photoreceptors express Rh6, and Rh5 expression is absent.

It is likely that *otd* also acts during development of the larval optic interneurons, given the large expression domain of *otd* in the ocular segment. However, current data on the expression and function of *otd* in the developing larval optic lobe pioneer neurons and PDF-expressing lateral neurons are lacking.

### 4.2. Otd in the development of the *Drosophila* adult visual sensory system

The major visual sense organs of the adult fly are the compound eyes, which are generated postembryonically [40] and are distinct from the larval Bolwig's organ, which differentiates into a minor adult visual structure called the Hofbauer–Buchner eyelet [41]. Each of the compound eyes comprises approximately 800 individual units called ommatidia, and each ommatidium consists of eight photoreceptor cells. These photoreceptors project their axons into the optic lobes, which consist of four highly structured neuropiles (the lamina, medulla, lobula and lobula plate) that process and relay visual information to higher brain centres (figure 2*b*) [42]. The compound eyes develop from the eye-specific domain of the eye–antennal disc during early pupal life, and this domain is thought to derive from the ocular segment (figure 2*a*) [19]. Lineage analysis using mutants that delete various head segments suggests that the optic lobes also derive from the ocular segment [43].

Consistent with their origin from the ocular segment, these adult visual structures express the *otd* gene during their development. Thus, Otd is expressed in all of the developing photoreceptors of the compound eyes (and the Hofbauer–Buchner eyelet [38,44–46]). In the compound eye photoreceptors, *otd* is required for proper rhabdomere formation, as well as for the correct expression of Rh3, Rh5 and Rh6 rhodopsins (figure 2*c*) [46–48]. In the absence of *otd*, the photoreceptor rhabdomeres appear disorganized owing to a failure in the morphogenesis of these structures. Rh3 and Rh5 are totally eliminated, the domain of Rh6 expression expands widely, and Rh1 is ectopically expressed [46,47,49–51]. The *otd* gene has been shown to act in the initial specification of the optic lobe and may also be required in the central interneurons of the adult visual system [8,43]. However, currently there is little information on a later role of *otd* in the interneurons of the optic lobe or in the central targets of the ocellar photoreceptors, although higher centres in the protocerebrum involved in visual processing and memory are affected by the loss-of-function of *otd* [12].

In summary, the cephalic gap gene *otd* is required for the development of larval and adult visual sense organs. Moreover, it may also play important roles in the development of the optic lobe and its visual interneurons.

### 4.3. The mouse visual circuit, which shares similarities with flies, requires the otd homologues, *Otx1/2*, for development

As noted by Cajal a century ago, there are remarkable similarities in the visual circuits of insects and mammals [52]. Furthermore, recent cellular and molecular studies indicate that these circuits are based on common design principles in the two animal groups [42]. Thus, as in *Drosophila*, in the mouse visual system, different types of photoreceptor cells project in parallel to a small number of interneuronal types. Moreover, these interneurons are arranged in an ordered manner in multiple parallel cell layers, which are interconnected orthogonally such that spatial relationships are retinotopically preserved across layers (figure 2*b*).

In addition to these similarities in circuit organization, there are parallels in the molecular mechanisms of visual system development in fly and mouse, and these are exemplified by the comparable expression and function of *otd* and its mammalian homologues *Otx1/2* [53,54]. In early mouse embryogenesis, *Otx1* and *Otx2* are expressed in the precursors of developing sensory organs such as the optic vesicle and the otic vesicle, as well as in the anlagen of the forebrain. Both genes are expressed throughout the optic vesicle at early stages; subsequently, their expression becomes more regionalized. *Otx1* continues to be transcribed later in development in the iris, ciliary process and lachrymal gland, whereas Otx2 becomes restricted to the dorsal part of the optic vesicle and the presumptive retinal pigment epithelium territory [24,53,55,56]. Later in embryogenesis, as the eye undergoes regional specification, a second wave of Otx2 expression appears in the neural retina in neuronal and glial precursors [55]. *Otx* genes are also expressed in higher brain centres associated with vision; the developing lateral geniculate and superior colliculus express *Otx1* and *Otx2*, and *Otx1* is expressed in layers 5 and 6 of the visual cortex as well [56,57].

Loss-of-function of *Otx* genes results in a variety of defects in the visual system. In *Otx1* null mice, the ciliary process is absent, the iris is reduced and the eye-associated glands do not differentiate [58]. *Otx2* null animals are early embryonic lethal owing to severe defects in gastrulation and head formation [59–61]. Allelic combinations of *Otx1* and *Otx2* that avoid early lethality reveal visual system defects such as the drastic reduction of the eyes, the malformation of the lens and retina, and the defasciculation of the retinal ganglion cell axons in the optic nerve (figure 2*c*) [58]. Conditional knockouts of *Otx2* during eye development result in a comparable reduction of the eye as well as a conversion of photoreceptors to amacrine cells [62]. Loss of *Otx1* in the visual cortex results in aberrant connectivity of cortical neurons with subcortical projections, suggesting a role for *Otx1* in the refinement of the cortical/subcortical circuitry [63].

Interestingly, Crx, which was identified as a member of the Otx family of transcription factors, was also shown to be specifically expressed in photoreceptor neurons in two phases [64,65]. During embryonic development, Crx is expressed in the cone photoreceptors, but it is most highly expressed postnatally in the differentiating rod photoreceptors [64]. Mice deficient for *Crx* function exhibit several defects in the retina. The proximal terminals of photoreceptor neurons fail to elaborate outer segment structures, a phenotype reminiscent of the defective rhabdomeres of *otd* null photoreceptors in *Drosophila* (figure 2*c*) [46,64]. The distal terminals of *Crx* null photoreceptors are also defective in that they are unable to initiate appropriate synaptogenesis in the outer plexiform layer [66]. Furthermore, there appears to be a degeneration of photoreceptor neurons, as evidenced by the progressive loss of nuclei from the nuclear layer of the retina [67]. A variety of *in vitro* and *in vivo* assays have demonstrated that Crx regulates the expression of many photoreceptor genes [65,68,69]. *Crx* null mice have a higher level of spontaneous activity of the bipolar cells in the retina, resulting in an increased synaptogenesis between the bipolar cells and the retinal ganglion cells [70].

In summary, similar to the fly *otd* gene, the mammalian *Otx1/2* genes are expressed in the developing peripheral and central visual systems, and are crucial for their correct development.

### 4.4. Coincidence, causality or conservation?

In this review, we have highlighted the functional similarities in the pervasive requirement of the *ems/Emx* genes of flies and mice in the development of the olfactory sensory systems of these animals, which share striking morphological similarities. Thus, the fly *ems* acts in the development of the larval peripheral and central olfactory system, and the adult peripheral and central olfactory system; the mouse homologues of *ems*, *Emx1/2*, act in the development of the mouse peripheral olfactory system, as well as in the mouse central olfactory system. Moreover, similar to the pervasive requirement of *ems/Emx* genes in olfactory system development, the visual sensory systems of flies and mice, which also share striking structural similarities, require the action of another cephalic gap gene, *otd*, and its mouse homologues, *Otx1/2* (and *Crx*). Thus, the fly *otd* acts in the development of the larval peripheral and central visual system, and the adult peripheral and central visual system; the mouse homologues of *otd*, *Otx1/2*, act in the development of the mouse peripheral visual system, as well as in the mouse central visual system. Are these similarities purely coincidental, or is there a developmental or evolutionary explanation behind them?

Developmental control genes have been shown to act recurrently in many related and unrelated developmental processes and contexts, and hence the possibility that these similarities are purely coincidental cannot be completely ruled out. However, a more reasonable view is that these remarkable similarities in cephalic gap gene action in the construction of two sensory systems that are structurally so similar are not purely coincidental.

One possible explanation for the striking similarities in cephalic gap gene action, emanating from the latter view, is that they reflect lineage relationships. During early embryonic development in insects and mammals, both *ems/Emx* and *otd/Otx* genes act in large, evolutionarily conserved domains in the anterior cephalic neuroectoderm. The neural cells that derive lineally from these cephalic domains (and hence fate-map to these domains) may continue to require the cephalic gap gene during their subsequent development. For example, in *Drosophila*, peripheral and central elements of the larval and adult olfactory system are generated by precursors that derive from the Ems-expressing antennal segment, and many of these elements continue to require the *ems* gene reiteratively during their embryonic and postembryonic development. Thus, the recurrent utilization of *ems* during the development of the fly olfactory system might merely be a manifestation of the developmental history of the cells that compose the olfactory system. Similar considerations hold for the *otd* gene in fly visual system development, as well as for the *Emx* and *Otx* genes in mammalian olfactory and visual system development. Interestingly, the fact that both peripheral and central elements of a given sensory system require the same cephalic gap gene could serve to genetically couple the development of the sense organs and their central brain circuitry such that both evolve in a coordinated manner.

There is also another possible explanation consistent with the conserved uses of genes during development as outlined earlier. The remarkable similarities in cephalic gap gene expression and function during the development of sensory systems in both flies and mammals could be a reflection of a common evolutionary origin of these sensory systems. Thus, it is possible that the sensory systems of extant insects and vertebrates have evolved from sensory systems that were already present in the last urbilaterian ancestor of insects and vertebrates. While this hypothesis needs careful testing, there is some evidence to support it. Recent work has shown that expression and function of many of the developmental control genes involved in anteroposterior and dorsoventral patterning of the nervous system, including the cephalic gap genes, are conserved in invertebrates and vertebrates [71–73]. Moreover, there is increasing evidence that specific neuronal types, and even complex associative brain areas might be conserved among bilaterian animals [74–77]. These findings suggest that conserved developmental control genes and patterning mechanisms might already have been present in the last common urbilaterian ancestor, and these in turn could have given rise to a reasonably complex nervous system over 600 Myr ago [78]. In this scenario, the similarities in the development of sensory systems in insects and mammals that we have highlighted here might reflect their common origin from ancestral sensory systems with comparable developmental features. If this is the case, then the strikingly similar organization of the olfactory and visual circuitry in insects and mammals might be due, at least in part, to a common evolutionary origin from ancestral olfactory and visual systems, which arose from embryonic domains that expressed ancestral homologues of *ems/Emx* and *otd/Otx*.

## 5. Acknowledgements

This work was supported by grants from NCBS-TIFR, Department of Biotechnology, Government of India, the Indo Swiss Bilateral Research Initiative and the Swiss NSF. We thank the Department of Science and Technology, Government of India—Centre for Nanotechnology (no. SR/S5/NM-36/2005) and Central Imaging and Flow Cytometry Facility.

## References

[1] Dalton D Chadwick R McGinnis W 1989 Expression and embryonic function of empty spiracles: a *Drosophila* homeo box gene with two patterning functions on the anterior–posterior axis of the embryo. Genes Dev. 3, 1940–1956 10.1101/gad.3.12a.1940 (doi:10.1101/gad.3.12a.1940) 2576012

[2] Finkelstein R Perrimon N 1990 The orthodenticle gene is regulated by bicoid and torso and specifies *Drosophila* head development. Nature 346, 485–488 10.1038/346485a0 (doi:10.1038/346485a0) 1974036

[3] Walldorf U Gehring WJ 1992 Empty spiracles, a gap gene containing a homeobox involved in *Drosophila* head development. EMBO J. 11, 2247–2259 1376248 10.1002/j.1460-2075.1992.tb05284.x PMC556692

[4] Jurgens G Hartenstein V 1993 The terminal regions of the body pattern. In The development of Drosophila melanogaster, vol. 1 (eds Bate M Martinez-Arias A), pp. 687–746 Plainview, NY: Cold Spring Harbor Laboratory Press

[5] Gao Q Finkelstein R 1998 Targeting gene expression to the head: the *Drosophila* orthodenticle gene is a direct target of the Bicoid morphogen. Development 125, 4185–4193 9753673 10.1242/dev.125.21.4185

[6] Cohen SM Jürgens G 1990 Mediation of *Drosophila* head development by gap-like segmentation genes. Nature 346, 482–485 10.1038/346482a0 (doi:10.1038/346482a0) 1974035

[7] Finkelstein R Smouse D Capaci TM Spradling AC Perrimon N 1990 The orthodenticle gene encodes a novel homeo domain protein involved in the development of the *Drosophila* nervous system and ocellar visual structures. Genes Dev. 4, 1516–1527 10.1101/gad.4.9.1516 (doi:10.1101/gad.4.9.1516) 1979296

[8] Schmidt-Ott U González-Gaitán M Jäckle H Technau GM 1994 Number, identity, and sequence of the *Drosophila* head segments as revealed by neural elements and their deletion patterns in mutants. Proc. Natl Acad. Sci. USA 91, 8363–8367 10.1073/pnas.91.18.8363 (doi:10.1073/pnas.91.18.8363) 7915837 PMC44606

[9] White K Kankel DR 1978 Patterns of cell division and cell movement in the formation of the imaginal nervous system in *Drosophila* melanogaster. Dev. Biol. 65, 296–321 10.1016/0012-1606(78)90029-5 (doi:10.1016/0012-1606(78)90029-5) 98369

[10] Hartenstein V Campos-Ortega JA 1984 Early neurogenesis in wild-type *Drosophila melanogaster*. Dev. Genes Evol. 193, 308–325 10.1007/BF00848159 28305340

[11] Stocker RF 2008 Design of the larval chemosensory system. Adv. Exp. Med. Biol. 628, 69–81 10.1007/978-0-387-78261-4_5 (doi:10.1007/978-0-387-78261-4_5) 18683639

[12] Hirth F Therianos S Loop T Gehring WJ Reichert H Furukubo-Tokunaga K 1995 Developmental defects in brain segmentation caused by mutations of the homeobox genes orthodenticle and empty spiracles in *Drosophila*. Neuron 15, 769–778 10.1016/0896-6273(95)90169-8 (doi:10.1016/0896-6273(95)90169-8) 7576627

[13] Lee T Luo L 2001 Mosaic analysis with a repressible cell marker (MARCM) for *Drosophila* neural development. Trends Neurosci. 24, 251–254 10.1016/S0166-2236(00)01791-4 (doi:10.1016/S0166-2236(00)01791-4) 11311363

[14] Rodrigues V Hummel T 2008 Development of the *Drosophila* olfactory system. Adv. Exp. Med. Biol. 628, 82–101 10.1007/978-0-387-78261-4_6 (doi:10.1007/978-0-387-78261-4_6) 18683640

[15] Jefferis GS Marin EC Stocker RF Luo L 2001 Target neuron prespecification in the olfactory map of *Drosophila*. Nature 414, 204–208 10.1038/35102574 (doi:10.1038/35102574) 11719930

[16] Das A Sen S Lichtneckert R Okada R Ito K Rodrigues V Reichert H 2008 *Drosophila* olfactory local interneurons and projection neurons derive from a common neuroblast lineage specified by the empty spiracles gene. Neural Dev. 3, 33 10.1186/1749-8104-3-33 (doi:10.1186/1749-8104-3-33) 19055770 PMC2647541

[17] Lai S-L Awasaki T Ito K Lee T 2008 Clonal analysis of *Drosophila* antennal lobe neurons: diverse neuronal architectures in the lateral neuroblast lineage. Development 135, 2883–2893 10.1242/dev.024380 (doi:10.1242/dev.024380) 18653555

[18] Marin EC Watts RJ Tanaka NK Ito K Luo L 2005 Developmentally programmed remodeling of the *Drosophila* olfactory circuit. Development 132, 725–737 10.1242/dev.01614 (doi:10.1242/dev.01614) 15659487

[19] Younossi-Hartenstein A Tepass U Hartenstein V 1993 Embryonic origin of the imaginal discs of the head of *Drosophila melanogaster*. Dev. Genes Evol. 203, 60–73 10.1007/BF00539891 (doi:10.1007/BF00539891) 28305981

[20] Lichtneckert R Nobs L Reichert H 2008 Empty spiracles is required for the development of olfactory projection neuron circuitry in *Drosophila*. Development 135, 2415–2424 10.1242/dev.022210 (doi:10.1242/dev.022210) 18550709

[21] Sen S Hartmann B Reichert H Rodrigues V 2010 Expression and function of the empty spiracles gene in olfactory sense organ development of *Drosophila* melanogaster. Development 137, 3687–3695 10.1242/dev.052407 (doi:10.1242/dev.052407) 20940227

[22] Hildebrand JG Shepherd GM 1997 Mechanisms of olfactory discrimination: converging evidence for common principles across phyla. Annu. Rev. Neurosci. 20, 595–631 10.1146/annurev.neuro.20.1.595 (doi:10.1146/annurev.neuro.20.1.595) 9056726

[23] Kay LM Stopfer M 2006 Information processing in the olfactory systems of insects and vertebrates. Semin. Cell Dev. Biol. 17, 433–442 10.1016/j.semcdb.2006.04.012 (doi:10.1016/j.semcdb.2006.04.012) 16766212

[24] Simeone A Acampora D Gulisano M Stornaiuolo A Boncinelli E 1992 Nested expression domains of four homeobox genes in developing rostral brain. Nature 358, 687–690 10.1038/358687a0 (doi:10.1038/358687a0) 1353865

[25] Simeone A Gulisano M Acampora D Stornaiuolo A Rambaldi M Boncinelli E 1992 Two vertebrate homeobox genes related to the *Drosophila* empty spiracles gene are expressed in the embryonic cerebral cortex. EMBO J. 11, 2541–2550 1352754 10.1002/j.1460-2075.1992.tb05319.x PMC556729

[26] Briata P Di Blas E Gulisano M Mallamaci A Iannone R Boncinelli E Corte G 1996 EMX1 homeoprotein is expressed in cell nuclei of the developing cerebral cortex and in the axons of the olfactory sensory neurons. Mech. Dev. 57, 169–180 10.1016/0925-4773(96)00544-8 (doi:10.1016/0925-4773(96)00544-8) 8843394

[27] Mallamaci A Iannone R Briata P Pintonello L Mercurio S Boncinelli E Corte G 1998 EMX2 protein in the developing mouse brain and olfactory area. Mech. Dev. 77, 165–172 10.1016/S0925-4773(98)00141-5 (doi:10.1016/S0925-4773(98)00141-5) 9831645

[28] Pellegrini M Mansouri A Simeone A Boncinelli E Gruss P 1996 Dentate gyrus formation requires Emx2. Development 122, 3893–3898 9012509 10.1242/dev.122.12.3893

[29] Yoshida M Suda Y Matsuo I Miyamoto N Takeda N Kuratani S Aizawa S 1997 Emx1 and Emx2 functions in development of dorsal telencephalon. Development 124, 101–111 9006071 10.1242/dev.124.1.101

[30] Cecchi C Boncinelli E 2000 *Emx* homeogenes and mouse brain development. Trends Neurosci. 23, 347–352 10.1016/S0166-2236(00)01608-8 (doi:10.1016/S0166-2236(00)01608-8) 10906797

[31] Bishop KM Garel S Nakagawa Y Rubenstein JLR O'Leary DDM 2003 Emx1 and Emx2 cooperate to regulate cortical size, lamination, neuronal differentiation, development of cortical efferents, and thalamocortical pathfinding. J. Comp. Neurol. 457, 345–360 10.1002/cne.10550 (doi:10.1002/cne.10550) 12561075

[32] Shinozaki K Yoshida M Nakamura M Aizawa S Suda Y 2004 Emx1 and Emx2 cooperate in initial phase of archipallium development. Mech. Dev. 121, 475–489 10.1016/j.mod.2004.03.013 (doi:10.1016/j.mod.2004.03.013) 15147765

[33] McIntyre JC Bose SC Stromberg AJ McClintock TS 2008 Emx2 stimulates odorant receptor gene expression. Chem. Senses 33, 825–837 10.1093/chemse/bjn061 (doi:10.1093/chemse/bjn061) 18854508 PMC2580733

[34] Bolwig N 1946 Senses and sense organs of the anterior end of the house fly larvæ. Copenhagen, Denmark: CA Reitzel

[35] Steller H Fischbach KF Rubin GM 1987 Disconnected: a locus required for neuronal pathway formation in the visual system of *Drosophila*. Cell 50, 1139–1153 10.1016/0092-8674(87)90180-2 (doi:10.1016/0092-8674(87)90180-2) 3113740

[36] Meinertzhagen IA 1973 Development of the compound eye and optic lobe of insects. In Developmental neurobiology of arthropods (ed. Young D), pp. 51–103 New York, NY: Cambridge University Press

[37] Green P Hartenstein AY Hartenstein V 1993 The embryonic development of the *Drosophila* visual system. Cell Tissue Res. 273, 583–598 10.1007/BF00333712 (doi:10.1007/BF00333712) 8402833

[38] Ranade SS Yang-Zhou D Kong SW McDonald EC Cook TA Pignoni F 2008 Analysis of the Otd-dependent transcriptome supports the evolutionary conservation of CRX/OTX/OTD functions in flies and vertebrates. Dev. Biol. 315, 521–534 10.1016/j.ydbio.2007.12.017 (doi:10.1016/j.ydbio.2007.12.017) 18241855 PMC2329912

[39] Sprecher SG Pichaud F Desplan C 2007 Adult and larval photoreceptors use different mechanisms to specify the same Rhodopsin fates. Genes Dev. 21, 2182–2195 10.1101/gad.1565407 (doi:10.1101/gad.1565407) 17785526 PMC1950857

[40] Wolff T Ready D 1993 Pattern formation in the *Drosophila* retina. In The development of Drosophila melanogaster, vol. 2 (eds Lawrence P Martinez AM), pp. 1277–1326 New York, NY: Cold Spring Harbor Laboratory Press

[41] Helfrich-Förster C Edwards T Yasuyama K Wisotzki B Schneuwly S Stanewsky R Meinertzhagen IA Hofbauer A 2002 The extraretinal eyelet of *Drosophila*: development, ultrastructure, and putative circadian function. J. Neurosci. 22, 9255–9266 12417651 10.1523/JNEUROSCI.22-21-09255.2002 PMC6758046

[42] Sanes JR Zipursky SL 2010 Design principles of insect and vertebrate visual systems. Neuron 66, 15–36 10.1016/j.neuron.2010.01.018 (doi:10.1016/j.neuron.2010.01.018) 20399726 PMC2871012

[43] Schmidt-Ott U González-Gaitán M Technau GM 1995 Analysis of neural elements in head-mutant *Drosophila* embryos suggests segmental origin of the optic lobes. Roux's Arch. Dev. Biol. 205, 31–44 10.1007/BF00188841 (doi:10.1007/BF00188841) 28306063

[44] Royet J Finkelstein R 1995 Pattern formation in *Drosophila* head development: the role of the orthodenticle homeobox gene. Development 121, 3561–3572 8582270 10.1242/dev.121.11.3561

[45] Royet J Finkelstein R 1997 Establishing primordia in the *Drosophila* eye–antennal imaginal disc: the roles of decapentaplegic, wingless and hedgehog. Development 124, 4793–4800 9428415 10.1242/dev.124.23.4793

[46] Vandendries ER Johnson D Reinke R 1996 Orthodenticle is required for photoreceptor cell development in the *Drosophila* eye. Dev. Biol. 173, 243–255 10.1006/dbio.1996.0020 (doi:10.1006/dbio.1996.0020) 8575625

[47] Tahayato A Sonneville R Pichaud F Wernet MF Papatsenko D Beaufils P Cook T Desplan C 2003 Otd/Crx, a dual regulator for the specification of ommatidia subtypes in the *Drosophila* retina. Dev. Cell 5, 391–402 10.1016/S1534-5807(03)00239-9 (doi:10.1016/S1534-5807(03)00239-9) 12967559

[48] Fichelson P Brigui A Pichaud F 2012 Orthodenticle and Kruppel homolog 1 regulate *Drosophila* photoreceptor maturation. Proc. Natl Acad. Sci. USA 109, 7893–7898 10.1073/pnas.1120276109 (doi:10.1073/pnas.1120276109) 22547825 PMC3356647

[49] McDonald EC Xie B Workman M Charlton-Perkins M Terrell DA Reischl J Wimmer EA Gebelein BA Cook TA 2010 Separable transcriptional regulatory domains within Otd control photoreceptor terminal differentiation events. Dev. Biol. 347, 122–132 10.1016/j.ydbio.2010.08.016 (doi:10.1016/j.ydbio.2010.08.016) 20732315 PMC2969183

[50] Mishra M Oke A Lebel C McDonald EC Plummer Z Cook TA Zelhof AC 2010 Pph13 and orthodenticle define a dual regulatory pathway for photoreceptor cell morphogenesis and function. Development 137, 2895–2904 10.1242/dev.051722 (doi:10.1242/dev.051722) 20667913 PMC2938920

[51] Johnston RJ Jr 2011 Interlocked feedforward loops control cell-type-specific Rhodopsin expression in the *Drosophila* eye. Cell 145, 956–968 10.1016/j.cell.2011.05.003 (doi:10.1016/j.cell.2011.05.003) 21663797 PMC3117217

[52] Cajal S Sanchez D 1915 Contribucion al conocimiento de los centros nerviosos del los insectos. Trab. Lab. Invest. Biol. 13, 1–167

[53] Simeone A Acampora D Mallamaci A Stornaiuolo A D'Apice MR Nigro V Boncinelli E 1993 A vertebrate gene related to orthodenticle contains a homeodomain of the bicoid class and demarcates anterior neuroectoderm in the gastrulating mouse embryo. EMBO J. 12, 2735–2747 8101484 10.1002/j.1460-2075.1993.tb05935.x PMC413524

[54] Acampora D Avantaggiato V Tuorto F Barone P Reichert H Finkelstein R Simeone A 1998 Murine Otx1 and *Drosophila* otd genes share conserved genetic functions required in invertebrate and vertebrate brain development. Development 125, 1691–1702 9521907 10.1242/dev.125.9.1691

[55] Bovolenta P Mallamaci A Briata P Corte G Boncinelli E 1997 Implication of OTX2 in pigment epithelium determination and neural retina differentiation. J. Neurosci. 17, 4243–4252 9151741 10.1523/JNEUROSCI.17-11-04243.1997 PMC6573571

[56] Acampora D Gulisano M Broccoli V Simeone A 2001 Otx genes in brain morphogenesis. Prog. Neurobiol. 64, 69–95 10.1016/S0301-0082(00)00042-3 (doi:10.1016/S0301-0082(00)00042-3) 11250063

[57] Frantz GD Weimann JM Levin ME McConnell SK 1994 Otx1 and Otx2 define layers and regions in developing cerebral cortex and cerebellum. J. Neurosci. 14, 5725–5740 7931541 10.1523/JNEUROSCI.14-10-05725.1994 PMC6577005

[58] Martinez-Morales JR Signore M Acampora D Simeone A Bovolenta P 2001 Otx genes are required for tissue specification in the developing eye. Development 128, 2019–2030 11493524 10.1242/dev.128.11.2019

[59] Acampora D Mazan S Lallemand Y Avantaggiato V Maury M Simeone A Brûlet P 1995 Forebrain and midbrain regions are deleted in Otx2^-/-^ mutants due to a defective anterior neuroectoderm specification during gastrulation. Development 121, 3279–3290 7588062 10.1242/dev.121.10.3279

[60] Matsuo I Kuratani S Kimura C Takeda N Aizawa S 1995 Mouse Otx2 functions in the formation and patterning of rostral head. Genes Dev. 9, 2646–2658 10.1101/gad.9.21.2646 (doi:10.1101/gad.9.21.2646) 7590242

[61] Ang SL Jin O Rhinn M Daigle N Stevenson L Rossant J 1996 A targeted mouse Otx2 mutation leads to severe defects in gastrulation and formation of axial mesoderm and to deletion of rostral brain. Development 122, 243–252 8565836 10.1242/dev.122.1.243

[62] Nishida A Furukawa A Koike C Tano Y Aizawa S Matsuo I Furukawa T, 2003 Otx2 homeobox gene controls retinal photoreceptor cell fate and pineal gland development. Nat. Neurosci. 6, 1255–1263 10.1038/nn1155 (doi:10.1038/nn1155) 14625556

[63] Weimann JM Zhang YA Levin ME Devine WP Brulet P McConnell SK 1999 Cortical neurons require Otx1 for the refinement of exuberant axonal projections to subcortical targets. Neuron 24, 819–831 10.1016/S0896-6273(00)81030-2 (doi:10.1016/S0896-6273(00)81030-2) 10624946

[64] Furukawa T Morrow EM Cepko CL 1997 Crx, a novel otx-like homeobox gene, shows photoreceptor-specific expression and regulates photoreceptor differentiation. Cell 91, 531–541 10.1016/S0092-8674(00)80439-0 (doi:10.1016/S0092-8674(00)80439-0) 9390562

[65] Chen S Wang Q-L Nie Z Sun H Lennon G Copeland NG Gilbert DJ Jenkins NA Zack DJ 1997 Crx, a novel Otx-like paired-homeodomain protein, binds to and transactivates photoreceptor cell-specific genes. Neuron 19, 1017–1030 10.1016/S0896-6273(00)80394-3 (doi:10.1016/S0896-6273(00)80394-3) 9390516

[66] Morrow EM Furukawa T Raviola E Cepko CL 2005 Synaptogenesis and outer segment formation are perturbed in the neural retina of Crx mutant mice. BMC Neurosci. 6, 5 10.1186/1471-2202-6-5 (doi:10.1186/1471-2202-6-5) 15676071 PMC548520

[67] Furukawa T Morrow EM Li T Davis FC Cepko CL 1999 Retinopathy and attenuated circadian entrainment in Crx-deficient mice. Nat. Genet. 23, 466–470 10.1038/70591 (doi:10.1038/70591) 10581037

[68] Corbo JC 2010 CRX ChIP-seq reveals the cis-regulatory architecture of mouse photoreceptors. Genome Res. 20, 1512–1525 10.1101/gr.109405.110 (doi:10.1101/gr.109405.110) 20693478 PMC2963815

[69] Livesey FJ Furukawa T Steffen MA Church GM Cepko CL 2000 Microarray analysis of the transcriptional network controlled by the photoreceptor homeobox gene Crx. Curr. Biol. 10, 301–310 10.1016/S0960-9822(00)00379-1 (doi:10.1016/S0960-9822(00)00379-1) 10744971

[70] Soto F Ma X Cecil JL Vo BQ Culican SM Kerschensteiner D 2012 Spontaneous activity promotes synapse formation in a cell-type-dependent manner in the developing retina. J. Neurosci. 32, 5426–5439 10.1523/JNEUROSCI.0194-12.2012 (doi:10.1523/JNEUROSCI.0194-12.2012) 22514306 PMC3353326

[71] Arendt D Nubler-Jung K 1999 Comparison of early nerve cord development in insects and vertebrates. Development 126, 2309–2325 10225991 10.1242/dev.126.11.2309

[72] Reichert H Simeone A 2001 Developmental genetic evidence for a monophyletic origin of the bilaterian brain. Phil. Trans. R. Soc. Lond. B 356, 1533–1544 10.1098/rstb.2001.0972 (doi:10.1098/rstb.2001.0972) 11604121 PMC1088534

[73] Lichtneckert R Reichert H 2008 Anteroposterior regionalization of the brain: genetic and comparative aspects. Adv. Exp. Med. Biol. 628, 32–41 10.1007/978-0-387-78261-4_2 (doi:10.1007/978-0-387-78261-4_2) 18683636

[74] Denes AS Jékely G Steinmetz PRH Raible F Snyman H Prud'homme B Ferrier DEK Balavoine G Arendt D 2007 Molecular architecture of annelid nerve cord supports common origin of nervous system centralization in bilateria. Cell 129, 277–288 10.1016/j.cell.2007.02.040 (doi:10.1016/j.cell.2007.02.040) 17448990

[75] Arendt D Denes AS Jékely G Tessmar-Raible K 2008 The evolution of nervous system centralization. Phil. Trans. R. Soc. B 363, 1523–1528 10.1098/rstb.2007.2242 (doi:10.1098/rstb.2007.2242) 18192182 PMC2614231

[76] Sweeney LB Luo L 2010 'Fore brain: a hint of the ancestral cortex. Cell 142, 679–681 10.1016/j.cell.2010.08.024 (doi:10.1016/j.cell.2010.08.024) 20813256

[77] Tomer R Denes AS Tessmar-Raible K Arendt D 2010 Profiling by image registration reveals common origin of annelid mushroom bodies and vertebrate pallium. Cell 142, 800–809 10.1016/j.cell.2010.07.043 (doi:10.1016/j.cell.2010.07.043) 20813265

[78] Peterson KJ Lyons JB Nowak KS Takacs CM Wargo MJ McPeek MA 2004 Estimating metazoan divergence times with a molecular clock. Proc. Natl Acad. Sci. USA 101, 6536–6541 10.1073/pnas.0401670101 (doi:10.1073/pnas.0401670101) 15084738 PMC404080

